# A comparison of machine learning models and Cox proportional hazards models regarding their ability to predict the risk of gastrointestinal cancer based on metabolic syndrome and its components

**DOI:** 10.3389/fonc.2023.1049787

**Published:** 2023-03-02

**Authors:** Tao Thi Tran, Jeonghee Lee, Madhawa Gunathilake, Junetae Kim, Sun-Young Kim, Hyunsoon Cho, Jeongseon Kim

**Affiliations:** ^1^ Department of Cancer Control and Population Health, Graduate School of Cancer Science and Policy, Goyang-si, Gyeonggi-do, Republic of Korea; ^2^ Department of Cancer Biomedical Science, Graduate School of Cancer Science and Policy, Goyang-si, Gyeonggi-do, Republic of Korea

**Keywords:** metabolic syndrome, gastrointestinal cancer, machine learning, prospective cohort study, Korea

## Abstract

**Background:**

Little is known about applying machine learning (ML) techniques to identify the important variables contributing to the occurrence of gastrointestinal (GI) cancer in epidemiological studies. We aimed to compare different ML models to a Cox proportional hazards (CPH) model regarding their ability to predict the risk of GI cancer based on metabolic syndrome (MetS) and its components.

**Methods:**

A total of 41,837 participants were included in a prospective cohort study. Incident cancer cases were identified by following up with participants until December 2019. We used CPH, random survival forest (RSF), survival trees (ST), gradient boosting (GB), survival support vector machine (SSVM), and extra survival trees (EST) models to explore the impact of MetS on GI cancer prediction. We used the C-index and integrated Brier score (IBS) to compare the models.

**Results:**

In all, 540 incident GI cancer cases were identified. The GB and SSVM models exhibited comparable performance to the CPH model concerning the C-index (0.725). We also recorded a similar IBS for all models (0.017). Fasting glucose and waist circumference were considered important predictors.

**Conclusions:**

Our study found comparably good performance concerning the C-index for the ML models and CPH model. This finding suggests that ML models may be considered another method for survival analysis when the CPH model’s conditions are not satisfied.

## Introduction

Gastrointestinal (GI) cancer refers to cancers affecting the digestive system. Gastric cancer, colorectal cancer, liver cancer, esophageal cancer, and pancreatic cancer are recognized as common GI cancers (1). According to an estimate in 2018, GI cancer accounted for 26% of new cancer cases and 35% of deaths related to cancer worldwide (1). The trend of GI cancers varies geographically by specific types; for instance, the highest rates of liver cancer, esophageal cancer, and gastric cancer are found in Asia (2). Although the trend has been upwards for colorectal and pancreatic cancer, the remaining cancers have experienced a downwards trend since 1999; GI cancer remains one of the most common cancers in Korea (3).

Although risk factors for specific types of GI cancer vary, lifestyle-related factors contribute significantly to the development of GI cancer (2). Specifically, a Western lifestyle has been documented to be associated with a higher prevalence of GI cancer (2). Notably, metabolic syndrome (MetS), which has been reported to be a global epidemic, might be considered an important mediator of the effect of a Western lifestyle on GI cancer development (2). MetS has been described as a group of conditions including obesity, hypertension, high blood sugar, and dyslipidemia (4). Currently, there are three definitions used for MetS diagnosis, namely, the WHO 1999, the National Cholesterol Education Program (NCEP) Adult Treatment Panel III (ATP III) 2005, and the International Diabetes Federation 2006 definitions. Although MetS definitions are modified by health care organizations for different regions, MetS remains a significant and alarming global public health problem (5).

Existing evidence from epidemiological studies has revealed that MetS may be an etiologic factor for GI cancer development. For example, a previous study of large-scale molecular data for 366,016 participants reported a potential link between MetS and an elevated risk of GI cancer regardless of the MetS definition used (6). Similarly, an increased risk of colorectal cancer, liver cancer, and gastric cancer was found in participants with MetS in other cohort studies (7–11). Notably, Cox proportional hazards (CPH) model was used in these studies; a CPH model is a linear regression with good interpretability (12). CPH model is known to be a semiparametric method in which survival times are assumed in relation to predictor variables in a particular way and proportional hazards (13).

To date, attention has been drawn to the application of machine learning (ML) to cancer prediction. In particular, the application of ML in survival analysis has been indicated in recent years (14, 15). However, the value of ML models compared to CPH model is ambiguous due to inconsistent results in previous studies (15–18). For example, an ML model outperformed a CPH model in predicting breast cancer survival in a previous study (16). In contrast, a comparably good performance was observed to predict survival in patients with oral and pharyngeal cancer in another study (17). Thus, the value of ML approaches compared to CPH model in survival analysis is still debatable.

To our knowledge, little is known about the application of ML techniques in epidemiological studies to identify important predictors affecting GI cancer development. In addition, no study has compared the accuracy of ML and CPH models for predicting GI cancer. Therefore, our study aimed to examine whether MetS and its components are related to GI cancer prediction and whether ML models outperform a CPH model in predicting GI cancer based on MetS and other confounders.

## Methods

### Study population

**Figure 1:**
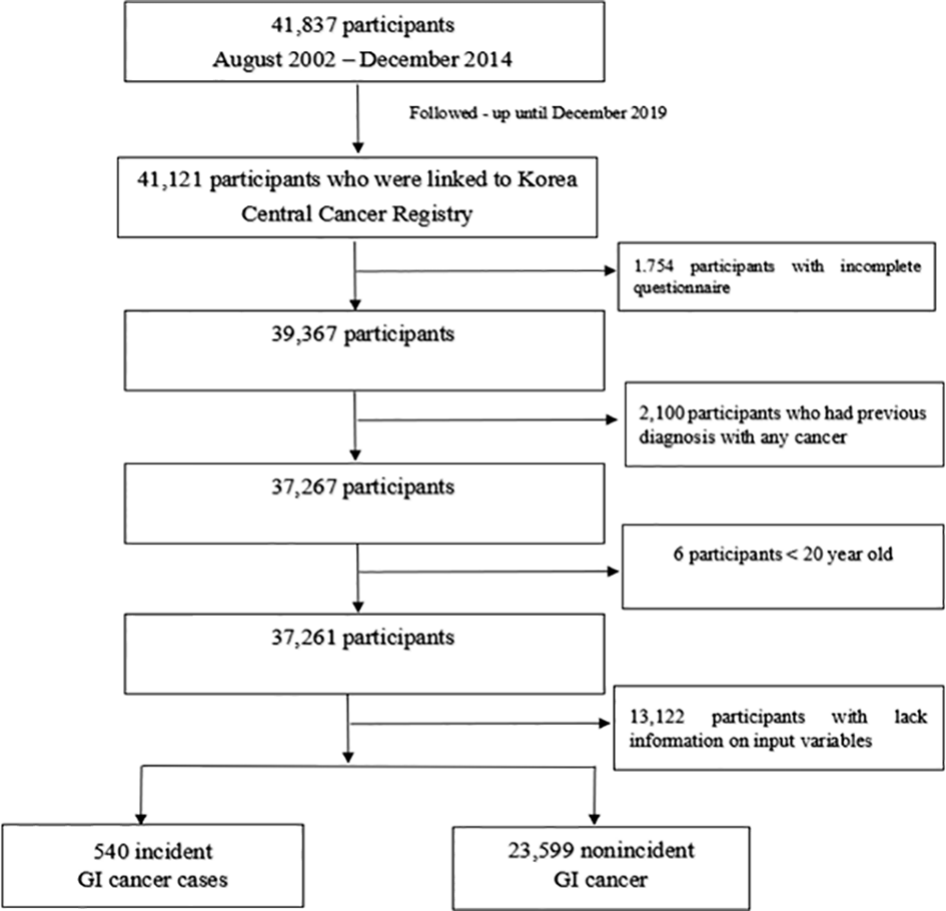
Flow chart of the study participants. Among 41,837 participants recruited, 41,121 participants were linked to the Korea Central Cancer Registry. Our final analysis included 24,139 participants after exclusion of 1,754 participants with incomplete questionnaires, 2,100 participants with a diagnosis of any cancer before recruitment, 6 participants aged <20 years, and 13,122 participants who lacked information on individual characteristics.

The Cancer Screenee Cohort Study was established in 2002 to explore the association of risk factors with cancer development in South Korea. The information of this cohort has been described (19). In brief, we recruited 41,837 participants aged 16 and older who visited the Center for Cancer Prevention and Detection at the National Cancer Center in South Korea for health examinations between August 2002 and December 2014. We required participants to complete baseline questionnaires and identified incident cancer cases by following up with participants until December 2019. Our final analysis included 24,139 participants after exclusion of 1,754 participants with incomplete questionnaires, 2,100 participants with a diagnosis of any cancer before recruitment, 6 participants aged <20 years, and 13,122 participants who lacked information on individual characteristics (**Figure 1**). We obtained written informed consent from all participants and approval for the study protocol from the institutional review board of the National Cancer Center (No. NCCNCS-07-077).

### Outcome and predictor measurement

Potential incident GI cancer cases were obtained by linking to the 2019 Korea National Cancer Incidence Database of the Korea Central Cancer Registry. We used the following International Classification of Diseases, 10th Revision codes to identify common incident GI cancers: gastric cancer (C16), colorectal cancer (C18-C20), liver cancer (C22), esophageal cancer (C15), gallbladder cancer (C23), and pancreatic cancer (C25). We identified 540 incident GI cancer cases, namely, 242 (44.8%) incident cases of gastric cancer, 123 (22.8%) incident cases of colorectal cancer, 102 (18.9%) incident cases of liver cancer, 41 (7.6%) incident cases of pancreatic cancer, 16 (3.0%) incident cases of gallbladder cancer, and 16 (3.0%) incident cases of esophageal cancer.

Currently, the MetS definition in Korea is based on the criteria of the ATP III of the NCEP and specific values of waist circumference from the World Health Organization and the Korean Society for the Study of Obesity. MetS was identified among those who met 3 or more of the following criteria (20):

1. Waist circumference: males ≥ 90 cm and females ≥85 cm
2. Blood pressure: ≥130/85 mmHg
3. Triglycerides: ≥150 mg/dL
4. High-density lipoprotein cholesterol (HDL cholesterol): males <40 mg/dL and females <50 mg/dL
5. Fasting glucose: ≥100 mg/dL or a history of diabetes.

We collected venous blood samples from participants at baseline after they had fasted for 8 hours to determine the blood-related components of MetS. Height (m) and weight (kg) were measured with InBody 3.0 (Biospace, Seoul, Korea) or automatic height and weight measurements (DS-102, Dong Shin Jenix Co., Ltd., Seoul, Korea). The measurement of waist circumference was performed with a tape measure 1 cm above the umbilicus with minimal respiration. A chemistry analyzer (TBA-200FR, Toshiba, Tokyo, Japan) was used to measure fasting glucose, triglyceride, and HDL cholesterol levels. Blood pressure was measured by trained personnel with an automatic blood pressure monitor (FT-200S, Jawon Medical, Kyungsan, Korea) after the patients had 15 minutes of rest (21). In addition, a self-administered questionnaire regarding information on baseline characteristics was completed by participants.

Predictors of GI cancer incidence in our study included MetS and its individual components (waist circumference, HDL, triglycerides, blood pressure, and fasting glucose). In addition, sociodemographic variables included age, sex, educational level (high school graduate or less and college or higher), marital status (married or cohabitating and others), monthly income (10,000 Korean won/month) (<200, 200-400, and >400), and first-degree family history of cancer (yes, no). Lifestyle factors included smoking status (nonsmoker, ex-smoker and current-smoker), alcohol consumption (nondrinker, former drinker and current-drinker), and physical activity (yes, no). These sociodemographic and lifestyle characteristics may be confounders for the association between MetS and GI cancer (6).

### Models and evaluation

We used a CPH model and ML survival models, including random survival forest (RSF), survival trees (ST), gradient boosting (GB), survival support vector machine (SSVM), and extra survival trees (EST), to predict GI cancer. The ability of these ML models to predict an outcome in right-censored time-to-event data has been documented in the literature as follows:

Random forest is an ML model that is most frequently used to solve problems in relation to classification and regression by constructing ensembles from decision trees and combining results to give a final decision. RSF extends random forest to censored lifetime data (22).

ST is another forest approach that has been widely used to handle time-to-event data. The implementation of ST is as follows: data partitioning is performed based on a criterion for splitting, and objects with similar events are grouped as the same node. (23).

Similar to RSF, the GB model is an ensemble model that combines the predictions of multiple base learners to improve the prediction of the overall model. However, RSF averages predictions from independent trees to obtain the overall prediction, while the GB model is additive (24).

Support vector machine aims to find a hyperplane to maximize the margin between classes. SSVM is an extension of the support vector machine to handle time-to-event data (16).

The EST model is a slightly different version of RSF (25). In comparison with RSF, the splitting criteria of EST are more random (26).

We used the following two evaluation metrics, which have been widely used in survival analyses in the literature, to compare the performance of the regression models (15, 22, 23, 26). The concordance index (C-index) is a rank order statistic for predictions against true outcomes and is defined as the ratio of the concordant pairs to the total comparable pairs (23); the closer the C-index is to 1, the better the model performs (15, 26). The integrated Brier score (IBS) reflects calibration over all time points, with a smaller value indicating greater accuracy (22). Furthermore, we evaluated the models based on the time-dependent area under the curve (AUC).

### Statistical analysis

We calculated person-years from baseline to the date of cancer diagnosis, death, or end of follow-up (December 31, 2019), whichever came first. We used chi-square tests and Wilcoxon rank-sum tests to compare the baseline characteristics between the incident GI cancer cases and nonincident GI cancer.

There were several steps for model development. First, we used an 80:20 ratio to randomly split the data into training and testing datasets. The purposes of the training and testing datasets were to fit the model and evaluate the final model, respectively. Second, a grid search was utilized to search hyperparameters for C-index maximization with 10-fold cross validation. We found the following optimal hyperparameters: n_estimators=400, max_depth=4 for RSF, max_depth=4 for ST, learning_rate=1 and max_depth=1 for GB, alpha=0.0002 for SSVM, and n_estimators=500, max_depth=4 for EST. Third, we fit the models using the training dataset based on selected input variables, the optimal hyperparameters, and default values of other hyperparameters. Fourth, the testing dataset was used to evaluate and compare model performance. Then, the ELI5 package was used to explore the contribution of predictors to the models, which calculates important variables based on the permutation important method by identifying the weight of variables (26). We used bootstrapping and 10-fold cross validation to assess the robustness of the models.

Furthermore, we used the CPH model to explore the specific associations of MetS and its components with incident GI cancer after adjusting for the aforementioned confounding factors. We performed statistical analyses by using Python software (version 3.7.9) with the scikit-survival library (26) and SAS software (version 9.4, SAS Institute, Cary, NC, USA) with a two-sided *P* value less than 0.05 was considered statistically significant.

## Results

### Characteristics of the participants

A total of 540 incident GI cancer cases were identified among 24,139 participants during the follow-up period (mean, 10.7 years), among which gastric cancer accounted for the highest number of cases (44.8%), followed by colorectal cancer (22.8%). Compared with the nonincident GI cancer group, the incident GI cancer group comprised older patients (54.7 ± 8.9 years old vs. 49.2 ± 9.1 years old, P<0.001) and patients who exhibited higher proportions of a low education level (49.4% vs. 44.1%, P=0.013), a low income level (23.9% vs. 15.0%, P<0.001), being married or cohabitating (93.7% vs. 90.6%, P=0.015), a first-degree family history of cancer (49.6% vs. 44.3%, P=0.014), and BMI≥25 (44.3% vs. 33.5%, P<0.001). In addition, this group tended to be current smokers (30.0% vs. 25.0%, P<0.001) and current drinkers (67.8% vs. 65.4%, P<0.001). With regard to MetS, incident GI cancer cases accounted for a higher proportion of MetS than those without incident GI cancer (26.3% vs. 18.2%, P<0.001). Similarly, the proportions of central obesity, elevated blood pressure, and high fasting glucose levels tended to be higher in incident GI cancer cases than in nonincident GI cancer (45.2% vs. 34.6%, P<0.001, 48.5% vs. 37.5%, P<0001, and 32.2% vs. 19.2%, P<0.001, respectively) (**Table 1**).

**Table 1:** Characteristics of the study subjects.

Characteristics	Total		
	Nonincident GI cancer (n=23,599)	Incident GI cancer (n=540)	*P* value^a^
Age^b^	49.2 ± 9.1	54.7 ± 8.9	<0.001
Follow-up duration (year)^b^	10.8 ± 3.8	6.1 ± 4.5	<0.001
Sex (n, %)			
Male	12971 (55.0)	400 (74.1)	<0.001
Female	10628 (45.0)	140 (25.9)	
Educational level (n, %)			
High school graduate or less	10405 (44.1)	267 (49.4)	0.013
College or higher	13194 (55.9)	273 (50.6)	
Household income (10,000 won/month) (n, %)			
<200	3539 (15.0)	129 (23.9)	<0.001
200-400	7747 (32.8)	171 (31.7)	
>400	12313 (52.2)	240 (44.4)	
Marital status (n, %)			
Married or cohabitating	21390 (90.6)	506 (93.7)	0.015
Others	2209 (9.4)	34 (6.3)	
First-degree family history of cancer (n, %)			
Yes	10457 (44.3)	268 (49.6)	0.014
No	13142 (55.7)	272 (50.4)	
Smoking status (n, %)			
Nonsmokers	12217 (51.8)	200 (37.0)	<0.001
Ex-smokers	5472 (23.2)	178 (33.0)	
Current smokers	5910 (25.0)	162 (30.0)	
Alcohol consumption (n, %)			
Nondrinkers	6986 (29.6)	126 (23.3)	<0.001
Ex-drinkers	1175 (5.0)	48 (8.9)	
Current drinkers	15438 (65.4)	366 (67.8)	
Regular exercise (n, %)			
Yes	13342 (56.5)	329 (60.9)	0.042
No	10257 (43.5)	211 (39.1)	
BMI (kg/m^2^)			
<23	9377 (39.7)	141 (26.1)	<0.001
23-25	6321 (26.8)	160 (29.6)	
≥25	7901 (33.5)	239 (44.3)	
Metabolic syndrome (n, %)			
No	19305 (81.8)	398 (73.7)	<0.001
Yes	4294 (18.2)	142 (26.3)	
Waist circumference (n, %)			
Men <90 cm and women<85 cm	15439 (65.4)	296 (54.8)	<0.001
Men ≥90 cm and women≥85 cm	8160 (34.6)	244 (45.2)	
HDL (n, %)			
Men ≥40 mg/dL and women ≥50 mg/dL	19778 (83.8)	440 (81.5)	0.147
Men <40 mg/dL and women <50 mg/dL	3821 (16.2)	100 (18.5)	
Triglycerides (n, %)			
<150 mg/dL	17791 (75.4)	403 (74.6)	0.686
≥150 mg/dL	5808 (24.6)	137 (25.4)	
Blood pressure (n, %)			
<130/85 mmHg	14757 (62.5)	278 (51.5)	<0.001
≥130/85 mmHg	8842 (37.5)	262 (48.5)	
Fasting glucose (n, %)			
<100 mg/dL or no history of diabetes	19069 (80.8)	366 (67.8)	<0.001
≥100 mg/dL or history of diabetes	4530 (19.2)	174 (32.2)	

a Chi-square tests. We used chi-square tests and Wilcoxon rank-sum tests to compare the baseline characteristics between the incident GI cancer cases and nonincident GI cancer.

b values are presented as the mean ± SD.

### Model performance

**Figure 2:**
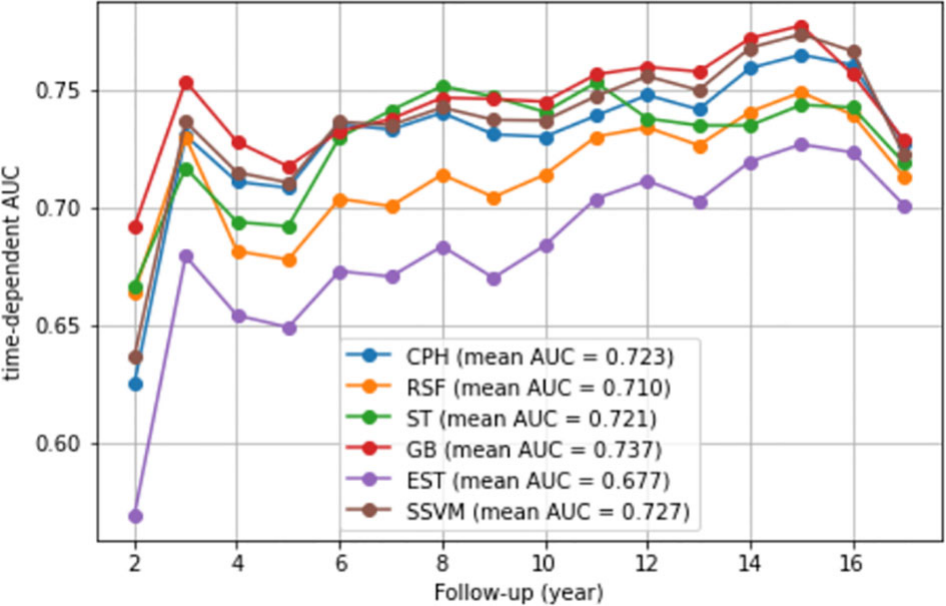
Time-dependent AUC. We presented the time-dependent AUC of the CPH model and five ML models, namely, the random survival forest, survival trees, gradient boosting, extra survival trees, and survival support vector machine models. The vertical axis is the time-dependent AUC. The horizontal axis is follow-up (year).

The C-index and IBS were used to compare the performance of the models. **Table 2** presents these metrics for the CPH and ML models based on the testing dataset. Comparably good performance was recorded for the GB, SSVM, and CPH models, with a C-index value of 0.725. The RSF and EST models exhibited a lower performance than the CPH model (0.699 vs. 0.725 and 0.671 vs. 0.725, respectively). IBS was not estimated for SSVM because it is applicable for models that can estimate a survival function. A similar value of IBS was found for the remaining models (0.017). Furthermore, comparably good discrimination was found for the ML and CPH models concerning the time-dependent AUC (**Figure 2**).

**Table 2:** C-index and integrated Brier score for the testing dataset.

Models	C-index on testing dataset	IBS
Cox	0.725 (0.723-0.727)	0.017
Random survival forest	0.699 (0.697-0.701)	0.017
Survival support vector machine	0.725 (0.723-0.727)	–
Survival trees	0.721 (0.719-0.723)	0.017
Gradient boosting	0.723 (0.721-0.725)	0.017
Extra survival trees	0.671 (0.668-0.673)	0.017

The C-index was estimated based on 100 bootstrapped data samples. The integrated Brier score (IBS) applies to models that can estimate a survival function. Thus, it is impossible to estimate the IBS for the survival support vector machine.

### Importance of predictors of GI cancer

**Table 3** presents the top five most important predictors of incident GI cancer based on the ML models. Notably, among the predictors related to MetS, fasting glucose was indicated as an important predictor for the occurrence of GI cancer across models. Furthermore, according to the GB model, waist circumference was one of the five important predictors contributing to incident GI cancer.

**Table 3:** Top 5 most important predictors for incident GI cancer based on the ML models.

Rank	RSF model		SSVM model		ST model		GB model		EST model.	
	Variables	Weight	Variables	Weight	Variables	Weight	Variables	Weight	Variables	Weight
1	Age	0.0975 ± 0.0365	Age	0.1472 ± 0.0494	Age	0.1822 ± 0.0614	Age	0.1784 ± 0.0531	Age	0.0562 ± 0.0214
2	Sex	0.0213 ± 0.0293	Sex	0.0158 ± 0.0197	Sex	0.0459 ± 0.0232	Sex	0.0336 ± 0.0217	Sex	0.0243 ± 0.0371
3	Smoking status	0.0029 ± 0.0059	First degree family history of cancer	0.0067 ± 0.0095	**Fasting glucose**	**0.0065 ± 0.0055**	**Fasting glucose**	**0.0030 ± 0.0065**	**Fasting glucose**	**0.0106 ± 0.0138**
4	**Fasting glucose**	**0.0029 ± 0.0104**	Smoking status	0.0054 ± 0.0091	Education	0.0037 ± 0.0068	**Waist circumference**	**0.0011 ± 0.0032**	Income	0.0045 ± 0.0068
5	First degree family history of cancer	0.0015 ± 0.0034	**Fasting glucose**	**0.0037 ± 0.0075**	Smoking status	0.0020 ± 0.0027	–	–	First degree family history of cancer	0.0012 ± 0.0045

Bold text indicates the predictors related to metabolic syndrome.

We then determined the specific relationships between MetS and its components and GI cancer development by using the CPH model. Notably, the important predictors were identified by the CPH model, which were similar to those of the ML models. In detail, a higher risk of GI cancer was found for participants with high waist circumference in both the crude model and adjusted model; the HRs (95% CIs) were 1.56 (1.32-1.85) for the former and 1.36 (1.15-1.62) for the latter. Similarly, high fasting glucose was observed in relation to an increased GI cancer risk in the crude model [HR=2.05 (1.71-2.46)], and this significance remained unchanged in the adjusted model [HR=1.41 (1.17-1.69)]. Furthermore, participants with MetS exhibited a significantly higher risk of GI cancer than those without MetS, and the HRs (95% CIs) were 1.64 (1.35-1.98) in the crude model and 1.29 (1.06-1.56) in the adjusted model (**Table 4**).

**Table 4:** Hazard ratios and 95% confidence intervals of incident GI related to metabolic syndrome and its components.

Exposure	Total			
	GI cancer cases	Person years	HR (95% CI) (Model 1)	HR (95% CI) (Model 2)
Waist circumference				
Men <90 cm, women <85 cm	296	168927.01	1.00	1.00
Men ≥90 cm, women ≥85 cm	244	89300.40	1.56 (1.32-1.85)	1.36 (1.15-1.62)
HDL				
Men ≥40 mg/dL, women ≥50 mg/dL	440	214258.72	1.00	1.00
Men <40 mg/dL, women <50 mg/dL	100	43968.68	1.10 (0.89-1.37)	1.11 (0.89-1.38)
Triglycerides				
<150 mg/dL	403	195275.75	1.00	1.00
≥150 mg/dL	137	62951.66	1.06 (0.87-1.28)	0.89 (0.73-1.09)
Blood pressure				
<130/85 mmHg	278	164464.34	1.00	1.00
≥130/85 mmHg	262	93763.07	1.65 (1.40-1.96)	1.26 (1.06-1.50)
Fasting glucose				
<100 mg/dL or no history of diabetes	366	209775.34	1.00	1.00
≥100 mg/dL or history of diabetes	174	48452.06	2.05 (1.71-2.46)	1.41 (1.17-1.69)
Metabolic syndrome				
No	398	212086.40	1.00	1.00
Yes	142	46141.00	1.64 (1.35-1.98)	1.29 (1.06-1.56)

HR, hazard ratio; CI, confidence interval.

Model 1: crude model; Model 2: adjusted for age, sex, education, alcohol consumption, marital status, smoking status, regular exercise, monthly income, and first-degree family history of cancer.

## Discussion

In this study, we constructed five ML models and compared them with a conventional CPH model to predict incident GI cancer and identify whether MetS and its components are potential predictors of GI cancer development. Our findings identified a comparably good performance concerning the C-index for the GB, SSVM, and CPH models. High fasting glucose was found to be a predictor for GI cancer development across six models. However, the important predictor was not restricted to high fasting glucose, and the importance of high waist circumference emerged in the GB and CPH models.

To date, attention has been drawn to the application of ML models to time-to-event data. As a result, many studies have been conducted to compare the predictive performance of ML models against CPH models. For example, based on a previous study, an extreme gradient boosting model outperformed a CPH model in predicting breast cancer survival based on C-index values (0.73 vs. 0.63) (16). Similar results were obtained in other studies comparing RSF models and CPH models for survival prediction in patients with liver transplantation or oral squamous cell carcinoma (18, 27). The C-index values obtained in these studies were 0.622/0.620 and 0.764/0.694 for the RSF and CPH models, respectively (18, 27). However, the value of ML models against CPH model is still open to discussion because inconsistent results have been obtained in other studies. Specifically, a comparably good performance was recorded for RSF, conditional inference forest, and CPH models in predicting the survival of patients with oral and pharyngeal cancer (17). Similarly, a CPH model showed better performance in another study conducted in China, where CPH and RSF models were used to predict the progression of high-grade glioma after proton and carbon ion radiotherapy (15).

To our knowledge, our study is the first attempt to use ML and CPH models for the prediction of GI cancer development based on MetS and other confounders. CPH model has been widely applied to investigate the impact of risk factors on incident cancers due to its simple, fast computation and meaningful outputs; however, its limitations need to be clarified (16). First, the proportional hazard assumption must be satisfied in the model. Survival curves for different strata need to have hazard functions that are proportional over time. Second, there is a linear relationship between log hazards and covariates (28). Thus, CPH model may not be appropriate for a dataset with nonlinearity due to a decreased accuracy in prediction (12, 17). To date, the development of ML has been documented to address the limitations of conventional statistical analysis in cancer prediction (16). The self-study, classification, prediction, and feature selection abilities of ML have been well recognized. ML methods can be adapted to deal with data with nonlinearity and high-dimensional covariates (22, 29). However, we found comparable performance for the CPH and ML models. This finding is consistent with some previous studies (17, 30) and may be explained as follows. First, a CPH model tries to fit the data to a specific model and tests the proportional hazards assumptions to examine the influence of predictors on an outcome (17). Thus, the proportional hazards assumption of CPH was satisfied in our data, which may be a potential explanation. Second, complex associations and interactions seem to be unimportant in our data. Third, a small number of predictors were used in our study. Overall, it is important to realize that the superiority of ML models is found only when a CPH model meets its limitations (17). Notably, we evaluated the models based on the C-index, which can account for censoring and does not depend on a single fixed evaluation time (27). Taken together, a CPH model should be considered a method in epidemiological studies when its conditions are satisfied. Additionally, a combination of ML and CPH models could be used to provide further insight into predictors; specifically, nonlinear interactions may be obtained using ML models, whereas a CPH model is used to summarize the risk in a dataset that is not suitable for CPH model analysis (14, 17).

Among the variables related to MetS, the importance of high fasting glucose as a predictor for GI cancer development was found across all models. This finding is consistent with the results of a previous study, where diabetes mellitus was documented to be associated with elevated GI cancer (31). Notably, a consistent association was also found for specific types of GI cancer. For instance, high fasting glucose was demonstrated to play an important role in gastric cancer development in our previous study (32). Similarly, we identified a positive association for colorectal cancer (11). Our finding was reinforced by the conclusions of other studies (33, 34). For example, a higher risk of colorectal cancer was observed in participants with type II diabetes in two large prospective cohorts in the U.S (33). Additionally, a higher risk of primary liver cancer and pancreatic cancer may be attributed to increased fasting glucose (35–37). Our study suggests that greater emphasis must be placed on participants with high fasting glucose, including those with prediabetes (100 mg/dL-125 mg/dL) and diabetes (≥126 mg/dL or a history of diabetes). Prediabetes may have a potential link to GI cancer development (32). Several biological mechanisms may be involved in the effect of high fasting glucose on GI cancer development. First, hyperglycemia could provide nutrients for tumor cells, which has certain effects on the proliferation of these cells. For example, epidermal growth factor expression and epidermal growth factor receptor transactivation may be induced by high glucose, which can contribute to promoting cell proliferation in pancreatic cancer (38). Second, there is a positive association between hyperglycemia and proinflammatory factor production; proinflammatory factors are known to stimulate the expression of oncogenes, regulate the cell cycle, promote the proliferation of tumor cells, inhibit apoptosis, and even induce the epithelial-to-mesenchymal transition (38). Third, insulin-like growth factor 1 (IGF-1) bioavailability could be promoted by insulin, which could inhibit apoptosis, stimulate cellular proliferation, and induce carcinogenesis (39).

Furthermore, high waist circumference was considered an important predictor of the occurrence of GI cancer in the CPH model and GB model in our study. Overall obesity has received more attention in previous studies than central obesity. However, central obesity may have more influence on cancer risk than overall obesity because metabolic derangement is reflected by insulin and IGF levels (40). This hypothesis was supported by a conclusion drawn from a previous study, which emphasized the stronger influence of waist circumference on colon cancer risk than BMI (41). Furthermore, the adverse effect of central obesity on GI cancer development was reinforced by evidence from two meta-analyses of prospective studies (42, 43). The pathophysiological mechanisms can be explained as follows. First, insulin resistance is an important mediator of the link between central obesity and cancer. In detail, high insulin levels lead to IGF activation, promote cellular proliferation, and inhibit apoptosis (44). Second, sex hormones may be a possible mechanism because they are related to a relationship between body size and shape. The pathogenesis of the link between body size and shape and cancer may include obesity-induced hypoxia, genetic susceptibility, and adipose stromal cell migration (44). Notably, MetS was demonstrated to be associated with GI cancer development in the CPH model. However, it was not indicated as the top five important variables in the ML models. A possible reason may be the stronger effects of high fasting glucose and waist circumference. These components are implied to be central factors for the causal link between MetS and GI cancer.

Notably, with regard to variable importance, CPH model exhibits a straightforward interpretation as an HR, whereas a large important variable has more influence on the transition to predict an outcome, and ML models do not provide the sign of the prediction (negative or positive effect) (45). To date, the direct comparison of CPH with ML models regarding interpretation is limited due to the lack of a common metric. Thus, it is necessary to address this limitation in further studies (18).

There are several strengths in our study. First, this is the first attempt to use an ML approach to predict and identify the adverse effects of high fasting glucose and central obesity on GI development with time-to-event data. Second, our study has a relatively large sample size with a long follow-up time, accurately identifying incident cases by linking to the national cancer registry using a high-quality database. Third, we used standardized operating procedures to perform the laboratory tests with standardized equipment and trained personnel. However, there are some limitations in our study. First, we used a small number of predictors. Second, the predictive power may be affected by a low proportion of incident cases in our study. Thus, further studies with a larger number of incident cases and predictors may be warranted to clarify the value of ML models against CPH model. Third, information on medication and dietary factors was not available to consider in our models.

In conclusion, our study found comparably good performance according to the C-index for the ML and CPH models. This finding suggested that ML models may be considered another method for survival analysis when a CPH model has limitations. However, further studies with a larger number of predictors are necessary to clarify the value of ML models. Furthermore, our study indicated that preventing high fasting glucose and central obesity could be expected to reduce GI cancer development.

## Data availability statement

The raw data supporting the conclusions of this article will be made available by the authors, without undue reservation.

## Ethics statement

The studies involving human participants were reviewed and approved by institutional review board of the National Cancer Center (No. NCCNCS-07-077). Written informed consent to participate in this study was provided by the participants’ legal guardian/next of kin.

## Author contributions

Formal analysis, TT, JL. Preparation of original draft, TT. Writing-review and editing, JSK, S-YK, HC, JTK, MG. Data curation, JL, JSK. Investigation, JL. Methodology, JL, JSK. Funding acquisition, JSK. Project administration, JSK. Supervision, JSK. All authors contributed to the article and approved the submitted version.
